# A simple technique for suspension and stabilization of retrieval bag and adnexa by anchoring to the abdominal wall

**DOI:** 10.1002/ccr3.5056

**Published:** 2021-11-19

**Authors:** Ioannis Chatzipapas, Nikolaos Kathopoulis, Konstantinos Kypriotis, Konstantinos Samartzis, Panagiota Siemou, Athanasios Protopapas

**Affiliations:** ^1^ 1^st^ Department of Obstetrics and Gynecology Alexandra Hospital National and Kapodistrian University of Athens Athens Greece; ^2^ Department of Radiology Alexandra Hospital Athens Greece

**Keywords:** cystectomy, endobag, laparoscopy

## Abstract

We describe a useful technique, in laparoscopic cystectomy in‐a‐bag, for suspension and stabilization of endobag and adnexa using temporary sutures. It intends to create an isolated field to avoid spillage of the cyst content into the abdomen in case of rupture, thereby allowing the safe laparoscopic removal of ovarian masses.

## Graphical abstract

This simple technique permits simultaneous suspension and stabilization of endobag and adnexa, facilitating the surgeon's work and making the operation safer for the patient.

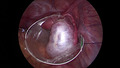

Laparoscopic surgery in the management of adnexal masses has become controversial regarding its oncological safety.^1^ Laparoscopic cystectomy in‐a‐bag is a technique proposed to manage suspicious adnexal cystic masses.^2^ In laparoscopic cystectomy in‐a‐bag operation, it is crucial that the endobag stays in place, to prevent spillage when inadvertent cyst's rupture occurs.^3^

We describe a useful technique, in laparoscopic cystectomy in‐a‐bag operation, for simultaneous suspension and stabilization of endobag and adnexa using temporary sutures that pass through the abdominal wall and endobag's ridge.

We insert a needle, 29‐mm curved Vicryl No. 0, percutaneously (Figure 1). It is pulled through the abdominal wall inside the body, passed under the brim of the endobag on its opposite sides, and then directed again to the abdominal wall, passed to the outside of the body (Figures 2, 3). The endobag used is retrieval bag (Grena). With this technique, the retrieval bag is suspended and therefore easily full‐deployed. Simultaneously, the cyst‐harboring adnexa is relatively stabilized by placing the suture string below it (Figure 4). Our technique permits suspension and stabilization of both bag and adnexa, facilitating the surgeon's work and making the operation safer for the patient.

**FIGURE 1:**
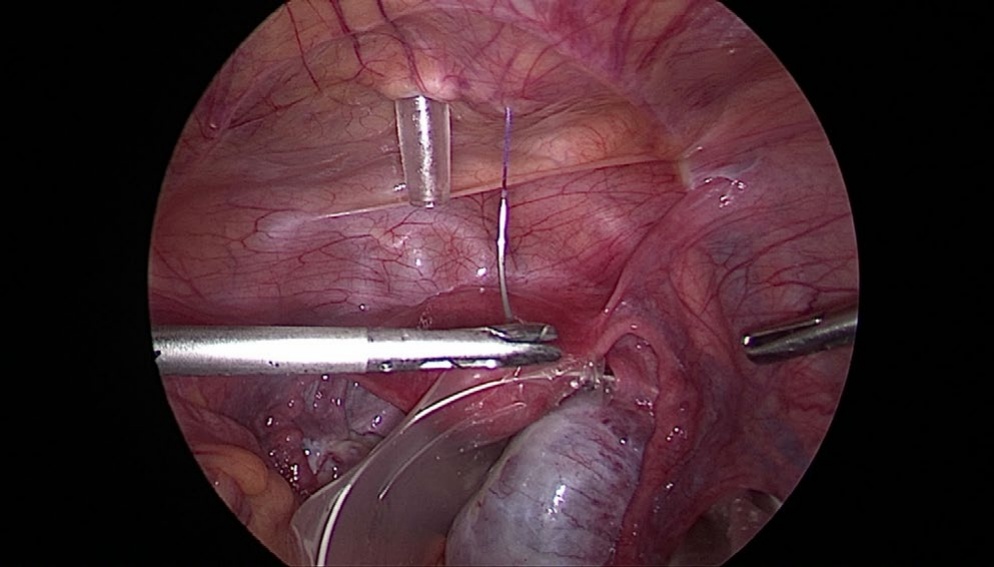
Insertion of the needle through abdominal wall into peritoneal cavity

**FIGURE 2:**
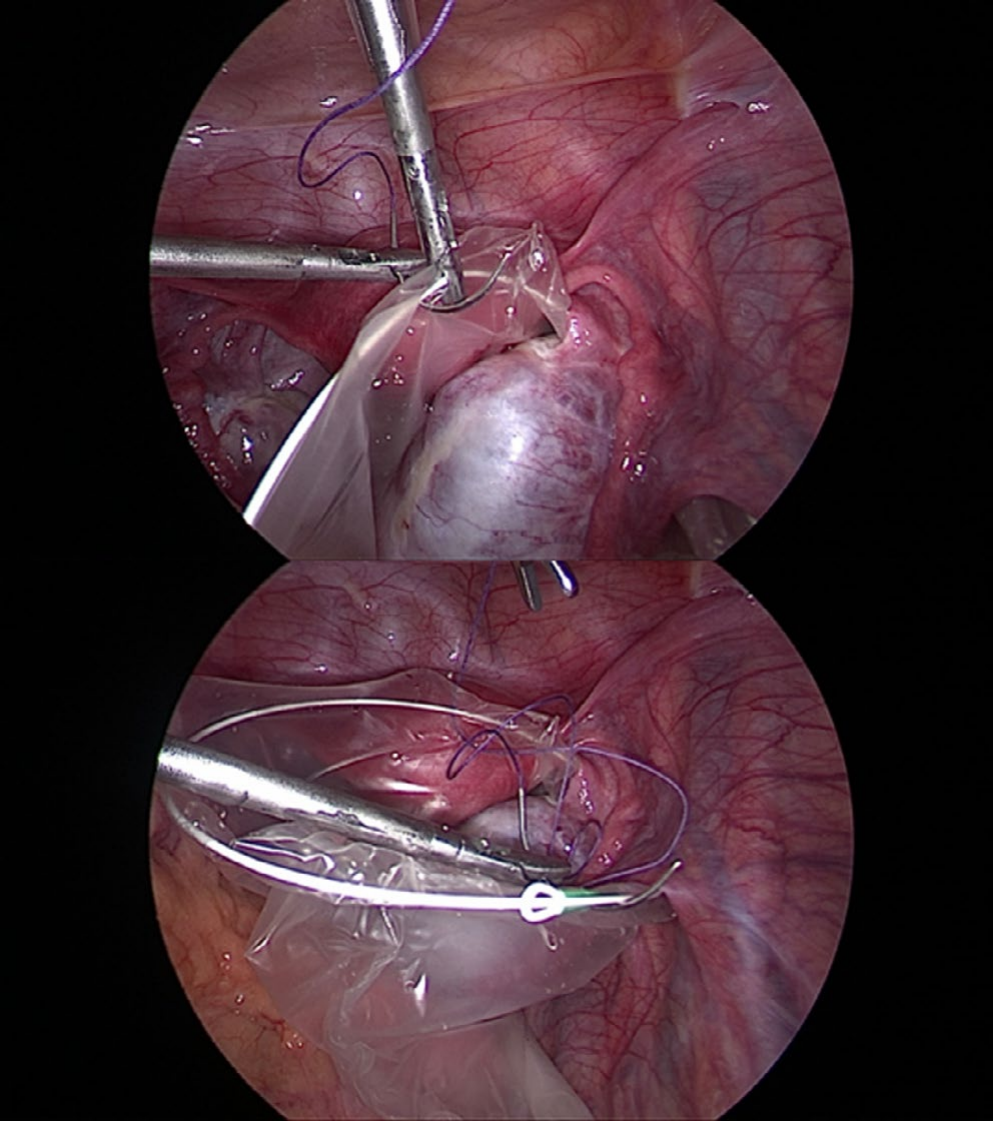
Suture passes under the brim of endobag on its opposite sides

**FIGURE 3:**
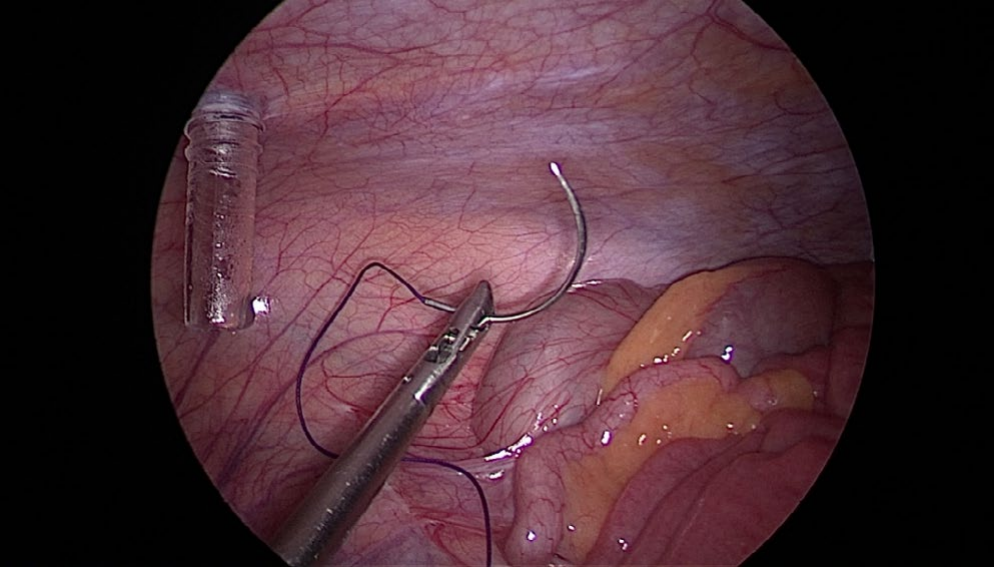
Extraction of the needle through abdominal wall outside of the body

**FIGURE 4:**
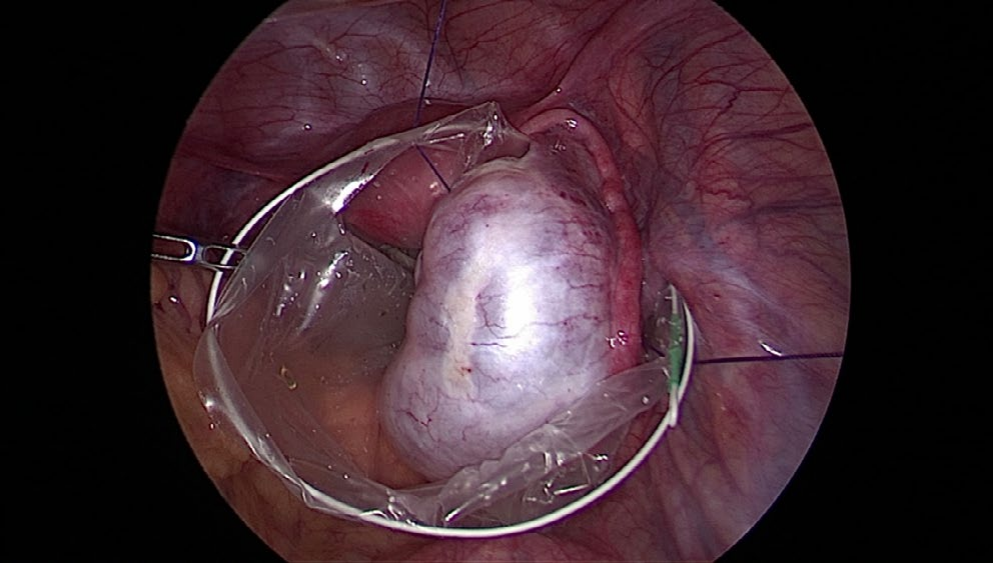
Endo‐peritoneal view

## 1 QUESTION

How can we make laparoscopic ovarian cystectomy a safer procedure?

## CONFLICT OF INTEREST

The authors declare no conflict of interest.

## AUTHOR CONTRIBUTIONS

IC: involved in conception and design, is a responsible surgeon. NK: wrote the manuscript. KK: is a responsible surgeon. KS, PS: collected and created the figures. AP: designed the project and edited the manuscript. All authors have read and approved the final manuscript.

## ETHICAL APPROVAL

The authors declare that written informed consent was obtained from the patient for publication of this case report. No patient‐identifying data have been released in the article.

## CONSENT

Published with written consent of the patient.
